# Influence of apocynin on cardiac remodeling in rats with streptozotocin-induced diabetes mellitus

**DOI:** 10.1186/s12933-017-0657-9

**Published:** 2018-01-17

**Authors:** R. Gimenes, C. Gimenes, C. M. Rosa, N. P. Xavier, D. H. S. Campos, A. A. H. Fernandes, M. D. M. Cezar, G. N. Guirado, L. U. Pagan, I. D. Chaer, D. C. Fernandes, F. R. Laurindo, A. C. Cicogna, M. P. Okoshi, K. Okoshi

**Affiliations:** 1 Department of Internal Medicine, Botucatu Medical School, Sao Paulo State University, UNESP, Botucatu, SP Brazil; 2 Sagrado Coração University, Bauru, SP Brazil; 3 Institute of Biosciences, Sao Paulo State University (UNESP), Botucatu, SP Brazil; 4 Department of Cardiopneumology, Medical School, Sao Paulo University, USP, São Paulo, Brazil; 5 Departamento de Clinica Medica, Faculdade de Medicina de Botucatu, Sao Paulo State University, UNESP, Rubiao Junior, S/N, Botucatu, SP CEP 18618-687 Brazil

**Keywords:** Diabetic cardiomyopathy, Heart failure, Oxidative stress, NADPH oxidase, NADPH oxidase blocker, Echocardiogram, Papillary muscle, Rat, Ventricular function, Myocardial function

## Abstract

**Background:**

Increased reactive oxygen species (ROS) generation in diabetes mellitus (DM) is an important mechanism leading to diabetic cardiomyopathy. Apocynin, a drug isolated from the herb *Picrorhiza kurroa*, is considered an antioxidant agent by inhibiting NADPH oxidase activity and improving ROS scavenging. This study analyzed the influence of apocynin on cardiac remodeling in diabetic rats.

**Methods:**

Six-month-old male Wistar rats were assigned into 4 groups: control (CTL, n = 15), control + apocynin (CTL + APO, n = 20), diabetes (DM, n = 20), and diabetes + apocynin (DM + APO, n = 20). DM was induced by streptozotocin. Seven days later, apocynin (16 mg/kg/day) or vehicle was initiated and maintained for 8 weeks. Left ventricular (LV) histological sections were used to analyze interstitial collagen fraction. NADPH oxidase activity was evaluated in LV samples. Comparisons between groups were performed by ANOVA for a 2 × 2 factorial design followed by the Bonferroni post hoc test.

**Results:**

Body weight (BW) was lower and glycemia higher in diabetic animals. Echocardiogram showed increased left atrial diameter, LV diastolic diameter, and LV mass indexed by BW in both diabetic groups; apocynin did not affect these indices. LV systolic function was impaired in DM groups and unchanged by apocynin. Isovolumic relaxation time was increased in DM groups; transmitral E/A ratio was higher in DM + APO compared to DM. Myocardial functional evaluation through papillary muscle preparations showed impaired contractile and relaxation function in both DM groups at baseline conditions. After positive inotropic stimulation, developed tension (DT) was lower in DM than CTL. In DM + APO, DT had values between those in DM and CTL + APO and did not significantly differ from either group. Myocardial interstitial collagen fraction was higher in DM than CTL and did not differ between DM + APO and CTL + APO. Serum activity of antioxidant enzymes glutathione peroxidase, superoxide dismutase (SOD), and catalase was lower in DM than CTL; apocynin restored catalase and SOD levels in DM + APO. Myocardial NADPH oxidase activity did not differ between groups.

**Conclusion:**

Apocynin restores serum antioxidant enzyme activity despite unchanged myocardial NADPH oxidase activity in diabetic rats.

## Background

Diabetes mellitus (DM) is considered an epidemic disease causing great challenge to public health all over the world. Aging, urbanization, and an unhealthy lifestyle such as inadequate diet and sedentarism are mainly responsible for its increased incidence [1]. Diabetes-induced hyperglycemia is related to damage in several organs and associated with vascular and cardiac diseases such as arterial hypertension, coronary artery disease, and heart failure, which are responsible for increased morbidity and mortality [2].

Diabetes-associated changes in myocardial structure and function, unrelated to coronary artery disease, arterial hypertension, valvular heart disease, or congenital heart disease, are called diabetic cardiomyopathy [3]. Several mechanisms are involved in diabetic cardiomyopathy including metabolic changes, myocyte hypertrophy, myocardial interstitial fibrosis, apoptosis, microvascular disease, autonomic dysfunction, energy production deterioration, intracellular calcium homeostasis alterations, and myocardial contractile proteins impairment [3–5]. Increase in reactive oxygen species (ROS) production caused by DM-induced metabolic changes is considered a major factor in triggering myocardial changes [6–8].

The nicotinamide adenine dinucleotide phosphate (NADPH) oxidase family is an important source of ROS in the cardiovascular system. NADPH oxidase activity is increased in cardiomyocytes and endothelial cells during cardiac hypertrophy induced by pressure overload contributing to ROS production and contractile dysfunction [9–11]. Studies in diabetic animals have shown elevated NADPH oxidase activity in the ventricular myocardium associated with cardiomyocyte hypertrophy and apoptosis, myocardial collagen deposition, and contractile dysfunction [12–15].

Apocynin, a drug isolated from the medicinal herb *Picrorhiza kurroa* [16], is considered an antioxidant agent which inhibits NADPH oxidase activity and improves ROS scavenging [17–19]. We have previously observed that apocynin reduces myocardial oxidative stress without changing NADPH oxidase activity in diabetic hypertensive rats [20]. Although beneficial effects of apocynin have been described in cardiac remodeling [11, 21–24], its cardiac effects have been poorly addressed in diabetic cardiomyopathy [13, 15, 25]. In this study, we investigated the influence of apocynin on cardiac remodeling in normotensive rats with streptozotocin-induced DM.

## Materials and methods

### Experimental groups

Six-month-old male Wistar rats were divided into four groups: control (CTL, n = 15); control treated with apocynin (CTL + APO, n = 20); diabetes (DM, n = 20); and diabetes treated with apocynin (DM + APO, n = 20). All animals were housed in a room under temperature control at 23 °C and kept on a 12-h light/dark cycle. Commercial chow and water were supplied ad libitum.

Diabetes was induced by intraperitoneal injection of streptozotocin (Sigma, St. Louis, MO, USA) at 50 mg/kg body weight diluted in 0.01 M citrate buffer pH 4.5 [26, 27]. Control groups received an intraperitoneal injection of vehicle only. Seven days after streptozotocin administration, blood glucose was measured by glucometer (Advantage®). Only rats with glycemia > 220 mg/dL were considered diabetic and included in the study [28]. Apocynin (Sigma, St. Louis, MO, USA) was added to drinking water at a dosage of 16 mg/kg/day for 8 weeks [20, 29]. Systolic blood pressure was measured at the end of experiment by the tail-cuff method using an electro-sphygmomanometer (*Narco Bio*-*System*^®^, *International Biomedical Inc*., USA) [30]. All rats underwent environment adaption before tail-cuff measurements.

### Echocardiographic study

Echocardiographic evaluation was performed at the end of the experimental period. We used a commercially available echocardiograph (General Electric Medical Systems, Vivid S6, Tirat Carmel, Israel) equipped with a 5–11.5 MHz multifrequency probe, as previously described [31–34]. Rats were anesthetized by intraperitoneal injection of a mixture of ketamine (50 mg/kg) and xylazine (0.5 mg/kg). Two-dimensionally guided M-mode images were obtained from short-axis views of the left ventricle (LV) just below the tip of the mitral-valve leaflets, and at the level of the aortic valve and left atrium. All LV structures were measured by the same observer (KO) and according to the leading-edge method of the American Society of Echocardiography [35]. Values obtained were the mean of at least five cardiac cycles on M-mode tracings. The following structural variables were measured: left atrial diameter (LA), LV diastolic (LVDD) and systolic (LVSD) diameter, and LV diastolic posterior wall thickness (PWT). LV mass was calculated using the formula [(LVDD + PWT + SWT)^3^ − (LVDD)^3^] × 1.04. LV function was assessed by the following parameters: endocardial fractional shortening (EFS), ejection fraction (EF), posterior wall shortening velocity (PWSV), tissue Doppler imaging (TDI) of mitral annulus systolic velocity (S′), early and late diastolic mitral inflow velocities (E and A waves), E/A ratio, isovolumic relaxation time (IVRT), TDI of early mitral annulus diastolic velocity (E′), and E/E′ ratio. Tissue Doppler variables (S′ and E′) were measured at the septal and lateral walls, and average values presented [36, 37].

### Myocardial functional evaluation

At the end of the experimental period, myocardial contractile performance was evaluated in isolated LV papillary muscle preparation as previously described [38–40]. Rats were anesthetized (pentobarbital sodium, 50 mg/kg, intraperitoneally) and decapitated. Hearts were quickly removed and placed in oxygenated Krebs–Henseleit solution at 28 °C. LV anterior or posterior papillary muscle was dissected free, mounted between two spring clips, and placed vertically in a chamber containing Krebs–Henseleit solution at 28 °C and oxygenated with a mixture of 95% O_2_ and 5% CO_2_ (pH 7.38). The composition of the Krebs–Henseleit solution in mM was as follows: 118.5 NaCl, 4.69 KCl, 1.25 CaCl_2_, 1.16 MgSO_4_, 1.18 KH_2_PO_4_, 5.50 glucose, and 25.88 NaHCO_3_. The spring clips were attached to a Kyowa model 120T-20B force transducer and a lever system which allowed for muscle length adjustment. Preparations were stimulated 12 times/min at a voltage 10% above threshold.

After a 60 min period, during which the preparations were permitted to shorten while carrying light loads, muscles were loaded to contract isometrically and stretched to the point where active tension development reached its maximum. After a 5 min period, during which preparations performed isotonic contractions, muscles were again placed under isometric conditions, and the peak of the length-tension curve (L_max_) was determined. A 15 min period of stable isometric contraction was imposed prior to the experimental period. One isometric contraction was then recorded for later analysis.

The following parameters were measured from the isometric contraction: peak of developed tension (DT), resting tension (RT), time to peak tension (TPT), maximum rate of tension development (+ dT/dt), and maximum rate of tension decline (− dT/dt). To evaluate myocardial contractile reserve, papillary muscle mechanical performance was evaluated after the following positive inotropic stimuli: 30 s post-rest contraction, extracellular Ca^2+^ concentration increase to 2.5 mM, and β-adrenergic agonist isoproterenol (10^−6^ M) added to the nutrient solution [41, 42]. Papillary muscle cross-sectional area (CSA) was calculated from muscle weight and length by assuming cylindrical uniformity and a specific gravity of 1.0. All force data were normalized for muscle CSA.

### Histologic analysis

Transverse LV sections were fixed in 10% buffered formalin and embedded in paraffin. Sections (5 µm thick) were stained with hematoxylin–eosin and collagen-specific stain picrosirius red (Sirius red F3BA in aqueous saturated picric acid) [43]. The smallest transverse diameters were measured in at least 50 myocytes from each LV, where the nucleus could be clearly identified [44]. On average, 20 microscopic fields were used to quantify interstitial collagen fraction. Perivascular collagen was excluded from this analysis [45]. Measurements were performed using a Leica microscope (magnification 40×) attached to a video camera and connected to a computer equipped with image analysis software (Image-Pro Plus 3.0, Media Cybernetics, Silver Spring, MD, USA).

### Plasma glucose and oxidative stress

After anesthesia and before heart removal from the chest, rats were decapitated and blood collected. Plasma was separated by centrifugation and used for quantification of glucose, glutathione peroxidase (GSH-Px), superoxide dismutase (SOD), and catalase.

Glutathione peroxidase (GSH-Px, E.C.1.11.1.9) activity was assayed in 15 μL of serum using 0.15 M phosphate buffer, pH 7.0, containing 5 mM EDTA, 0.1 mL of 0.0084 M NADPH, 4 μg of GSSG-reductase, 1.125 M sodium azide, and 0.15 M glutathione reduced form in a total volume of 0.3 mL [32]. Superoxide dismutase (SOD, E.C.1.15.1.1.) activity was determined in serum aliquots of 50 μL using its ability to inhibit reduction of nitroblue tetrazolium, in medium containing 50 mM phosphate buffer pH 7.4, 0.1 mM EDTA, 50 μM NBT, 78 μM NADH, and 3.3 μM phenazine methosulfate. One unit of superoxide dismutase was defined as the amount of protein needed to decrease the reference rate to 50% of maximum inhibition [28]. Catalase (E.C.1.11.1.6.) activity was evaluated in 50 mM phosphate buffer, pH 7.0, with 10 mM hydrogen peroxide. One unit of catalase was defined as the amount of enzyme needed to degrade 1 μmol H_2_O_2_ over 60 s, at 240 nm. Enzyme activity was analyzed at 25 °C using a microplate reader system (μQuant-MQX 200 with KC Junior software, Bio-TeK Instruments, Winooski, VT, USA). All reagents were purchased from Sigma (St. Louis, MO, USA).

### Myocardial NADPH oxidase activity

NADPH oxidase activity was evaluated in membrane-enriched cellular fraction by quantifying two dihydroethidium (DHE) oxidation-derived fluorescent compounds, 2-hydroxyethidium (EOH) and ethidium, by HPLC according to a previously described method [46, 47]. After washing cardiac tissue in phosphate buffered saline, muscle fragments (∼ 200 mg) were homogenized in 1 mL of ice-cold lysis buffer containing 50 mM Tris (pH 7.4), 100 mM DTPA, 0.1% β-mercaptoethanol, and protease inhibitors. The samples were sonicated (3 cycles of 10 s at 8 W) and centrifuged at 1000*g* for 3 min, at 4 °C. The supernatant was transferred to another microtube and centrifuged at 18,000*g* for 10 min at 4 °C. The supernatant was then centrifuged at 100,000*g* for 45 min, at 4 °C. The pellet was resuspended in 100 µL of lysis buffer. Total protein content was quantified by the Bradford method. Subsequently, 20 µg of membrane-enriched cellular fraction was incubated in phosphate buffer (50 mM, pH 7.4, with 0.1 mM DTPA) containing DHE (50 µM) and NADPH (300 µM) to a final volume of 100 µL, for 30 min at 37 °C in the dark. After adding 40 µL of 10% trichloroacetic acid, the samples were ice-cooled for 10 min in the dark, and centrifuged at 12,000*g* for 10 min at 4 °C. The supernatant was analyzed by HPLC and the fluorescent DHE-derived products were quantified.

### Statistical analysis

Results are expressed as mean and standard deviation or median and 25th and 75th percentiles according to normal or non-normal distribution, respectively. Variables were compared by analysis of variance (ANOVA) for a 2 × 2 factorial design followed by the Bonferroni post hoc test. Comparisons of interest: CTL + APO vs CTL, DM vs CTL, and DM + APO vs both CTL + APO and DM. Statistical significance was accepted at p < 0.05.

## Results

During the experimental period, 2 rats from CTL, 1 from CTL + APO, 2 from DM, and 3 from DM + APO groups died. One rat from DM and 1 from DM + APO did not have hyperglycemia and were excluded from the study. Initial and final body weights (BW) and systolic arterial pressure (SAP) are presented in Table 1. Initial BW did not differ between groups. Final BW was lower and SAP was higher in diabetic groups compared with their respective controls. Apocynin did not change BW or SAP.

**Table 1:** Body weight and systolic arterial pressure

	CTL (n = 13)	CTL + APO (n = 19)	DM (n = 17)	DM + APO (n = 16)
Initial BW (g)	460 (447–503)	461 (442–493)	467 (443–498)	469 (443–490)
Final BW (g)	543 ± 53	519 ± 52	316 ± 43*	313 ± 44^#^
SAP (mmHg)	118 ± 10	114 ± 12	129 ± 13*	131 ± 15^#^

Data are expressed as mean ± standard deviation or median and 25th and 75th percentiles

*CTL* control, *CTL* *+* *APO* control treated with apocynin, *DM* diabetes mellitus, *DM* *+* *APO* diabetes mellitus treated with apocynin, *BW* body weight, *SAP* systolic arterial pressure

* p < 0.05 vs CTL; ^#^ p < 0.05 vs CTL + APO

Table 2 shows echocardiographic structural data. As body weight significantly differed between groups, cardiac dimensions were normalized to its value to compensate for changes in body mass [48]. LVDD, LAD, and LVM indexed for BW were higher in diabetic groups compared to their respective controls. LV posterior wall thickness was lower in diabetics than controls. LVSD did not statistically differ between groups. Apocynin did not change cardiac structural data. Echocardiographic functional data are presented in Table 3. Consistent impairment of systolic function was observed in diabetic groups (EFS, EF, PWSV, and S′ were lower in both diabetic groups); apocynin did not change these variables. Isovolumic relaxation time was higher in diabetics than controls and unchanged by apocynin. Mitral E/A ratio was higher in DM + APO than DM.

**Table 2:** Echocardiographic structural data

	CTL (n = 13)	CTL + APO (n = 19)	DM (n = 17)	DM + APO (n = 16)
LVDD (mm)	8.39 ± 0.62	8.24 ± 0.49	8.03 ± 0.51	7.82 ± 0.65^#^
LVSD (mm)	3.92 ± 0.48	3.87 ± 0.62	4.11 ± 0.62	4.16 ± 0.58
LVDD/BW (mm/kg)	15.6 ± 2.15	16.0 ± 1.74	25.7 ± 2.87*	25.3 ± 3.13^#^
PWT (mm)	1.47 ± 0.07	1.47 ± 0.07	1.37 ± 0.08*	1.32 ± 0.08^#^
LA (mm)	5.76 ± 0.95	5.92 ± 0.63	5.68 ± 0.77	5.35 ± 0.81^#^
LA/BW (mm/kg)	10.8 ± 2.35	11.6 ± 1.91	18.1 ± 2.35*	17.3 ± 3.03^#^
LVM/BW (g/kg)	1.68 ± 0.27	1.71 ± 0.26	2.44 ± 0.32*	2.25 ± 0.32^#^

Data are expressed as mean ± standard deviation

*CTL* control, *CTL* *+* *APO* control treated with apocynin, *DM* diabetes mellitus, *DM* *+* *APO* diabetes mellitus treated with apocynin, *LVDD and LVSD* left ventricular (LV) diastolic and systolic diameters, respectively, *BW* body weight, *PWT* LV posterior wall thickness, *LA* left atrial diameter, *LVM* LV mass

* p < 0.05 vs CTL; ^#^ p < 0.05 vs CTL + APO

**Table 3:** Echocardiographic left ventricular functional data

	CTL (n = 13)	CTL + APO (n = 19)	DM (n = 17)	DM + APO (n = 16)
EFS (%)	53.3 ± 4.90	53.3 ± 5.45	49.0 ± 5.19*	46.9 ± 5.59^#^
EF	0.90 ± 0.03	0.89 ± 0.03	0.86 ± 0.04*	0.85 ± 0.05^#^
PWSV (mm/s)	38.1 ± 3.84	37.2 ± 3.51	27.9 ± 2.21*	30.2 ± 5.35^#^
S′ (cm/s)	3.50 (3.25–3.56)	3.50 (3.50–3.75)	3.00 (3.94–3.00)*	3.00 (2.50–3.00)^#^
Mitral E/A	1.46 ± 0.16	1.55 ± 0.28	1.29 ± 0.20	1.47 ± 0.30^§^
IVRT (ms)	28.9 ± 2.63	30.1 ± 3.12	38.6 ± 6.05*	38.6 ± 4.56^#^
E/E′	18.3 ± 3.51	18.8 ± 3.40	18.4 ± 3.01	19.7 ± 3.86

Data are expressed as mean ± standard deviation or median and 25th and 75th percentiles

*CTL* control, *CTL* *+* *APO* control treated with apocynin, *DM* diabetes mellitus, *DM* *+* *APO* diabetes mellitus treated with apocynin, *EFS* endocardial fractional shortening, *EF* ejection fraction, *PWSV* posterior wall shortening velocity, *S′* tissue Doppler imaging of systolic velocity of the mitral annulus (average of septal and lateral wall), *E/A* ratio between early (E)-to-late (A) diastolic mitral inflow, *IVRT* isovolumic relaxation time, *E′* tissue Doppler imaging of early diastolic velocity of mitral annulus (average of septal and lateral wall)

* p < 0.05 vs CTL; ^#^ p < 0.05 vs CTL + APO; ^§^ p < 0.05 vs DM

Baseline LV papillary muscle data are shown in Table 4. Diabetic groups had impaired contractile (lower + dT/dt and higher TPT) and relaxation (lower − dT/dt) function. Apocynin did not change basal papillary muscle performance. After all positive inotropic stimulation, DT was lower in DM than CTL. However, in DM + APO, DT values were between those in DM and CTL + APO and did not significantly differ from either group (Table 5).

**Table 4:** Basal left ventricular papillary muscle data

	CTL (n = 13)	CTL + APO (n = 13)	DM (n = 12)	DM + APO (n = 13)
DT (g/mm^2^)	7.53 ± 1.48	7.67 ± 1.20	5.98 ± 2.86	7.23 ± 1.82
+ dT/dt (g/mm^2^/s)	79.9 ± 15.4	78.5 ± 13.6	52.5 ± 25.5*	63.5 ± 16.5^#^
TPT (ms)	183 ± 13	194 ± 17	224 ± 16*	230 ± 13^#^
− dT/dt (g/mm^2^/s)	32.0 ± 6.17	31.3 ± 6.55	20.7 ± 8.11*	23.8 ± 5.84^#^
RT (g/mm^2^)	1.00 ± 0.31	0.86 ± 0.38	0.85 ± 0.47	0.79 ± 0.25

Data are expressed as mean ± standard deviation

*CTL* control, *CTL* *+* *APO* control treated with apocynin, *DM* diabetes mellitus, *DM* *+* *APO* diabetes mellitus treated with apocynin, *DT* peak of developed tension, *+* *dT/dt* maximum rate of tension development, *TPT* time to peak tension, *−* *dT/dt* maximum rate of tension decline, *RT* resting tension

* p < 0.05 vs CTL; ^#^ p < 0.05 vs CTL + APO

**Table 5:** Peak of developed tension (DT, g/mm^2^) of left ventricular papillary muscle following positive inotropic stimulation

	CTL (n = 13)	CTL + APO (n = 13)	DM (n = 12)	DM + APO (n = 13)
PP30	9.47 ± 2.36	9.44 ± 1.97	6.73 ± 3.23*	8.27 ± 2.29
[Ca^2+^]_o_ 2.5 mM	8.61 ± 2.14	8.95 ± 1.87	6.28 ± 2.94*	7.60 ± 2.11
Iso 10^−6^ M	7.79 ± 2.32	7.86 ± 1.63	5.62 ± 2.61*	6.54 ± 2.06

Data are expressed as mean ± standard deviation

*CTL* control, *CTL* *+* *APO* control treated with apocynin, *DM* diabetes mellitus, *DM* *+* *APO* diabetes mellitus treated with apocynin, *PP30* 30-s post-rest contraction, *[Ca*^*2+*^*]*_*o*_ *2.5* *mM* extracellular calcium concentration, *Iso 10*^*−6*^ *M* β-adrenergic agonist isoproterenol addition to the nutrient solution

* p < 0.05 vs CTL

Left ventricular myocyte diameters did not differ between groups. Myocardial interstitial collagen fraction was higher in DM than CTL (CTL 2.14 ± 1.29; CTL + APO 1.74 ± 0.90; DM 5.05 ± 2.98; DM + APO 3.12 ± 1.35%; p < 0.05 DM vs CTL). In DM + APO, values for this parameter were between those in DM and CTL + APO and did not significantly differ from either group.

Table 6 shows serum glucose levels and antioxidant enzyme activity. Glycaemia was higher in diabetic groups. Catalase was lower in DM than CTL. Superoxide dismutase and glutathione peroxidase were lower in DM and CTL + APO than CTL. In DM + APO, catalase was higher than DM and superoxide dismutase was higher than DM and CTL + APO. Myocardial NADPH oxidase activity did not differ between groups (Table 7).

**Table 6:** Glycemia and serum oxidative stress

	CTL (n = 8)	CTL + APO (n = 8)	DM (n = 8)	DM + APO (n = 8)
Glycemia (mg/dL)	100 ± 22	100 ± 16	599 ± 5*	589 ± 37^#^
Catalase (μmol/g)	4.21 ± 1.10	3.88 ± 1.40	1.08 ± 0.58*	4.43 ± 1.73^§^
SOD (nmol/mg)	6.43 ± 0.59	5.65 ± 0.56*	5.35 ± 0.28*	6.67 ± 0.80^#§^
GSH-Px (nmol/mg)	42.2 ± 7.70	25.0 ± 4.41*	30.9 ± 3.43*	29.5 ± 3.44

Data are expressed as mean ± standard deviation

*CTL* control, *CTL* *+* *APO* control treated with apocynin, *DM* diabetes mellitus, *DM* *+* *APO* diabetes mellitus treated with apocynin, *SOD* superoxide dismutase, *GSH-Px* glutathione peroxidase

* p < 0.05 vs CTL; ^#^ p < 0.05 vs CTL + APO; ^§^ p < 0.05 vs DM

**Table 7:** Myocardial NADPH oxidase activity

	CTL (n = 8)	CTL + APO (n = 8)	DM (n = 8)	DM + APO (n = 8)
EOH (nM)	38.1 ± 12.9	38.6 ± 11.1	36.0 ± 4.26	29.8 ± 6.38
Ethidium (nM)	103 ± 76	111 ± 77	102 ± 46	109 ± 51

Data are expressed as mean ± standard deviation. NADPH oxidase activity was evaluated in membrane-enriched cellular fraction by quantifying two dihydroethidium oxidation-derived fluorescent compounds, 2-hydroxyethidium (EOH) and ethidium

*CTL* control, *CTL* *+* *APO* control treated with apocynin, *DM* diabetes mellitus, *DM* *+* *APO* diabetes mellitus treated with apocynin

p = ns

## Discussion

Apocynin has been widely evaluated in experimental research as an antioxidant agent [17–19]. In literature several apocynin dosages have been used, ranging from 4 mg/kg/day [49] to 100 mg/kg/day [50]. In our study, we administered 16 mg/kg/day, a dosage usually reported in rodents [51, 52]. Streptozotocin has often been used to induce type 1 diabetes in rodents [28, 53]. As expected, diabetic rats presented higher glycemia levels and lower body weight than controls [26, 27, 54]. Apocynin did not attenuate hyperglycemia, body weight loss or blood pressure increase in diabetic groups.

Echocardiography showed changes compatible with diabetic cardiomyopathy [55, 56], characterized by dilation of the left chambers with systolic and diastolic dysfunction. Papillary muscle preparations are useful for evaluating myocardial function without influences from ventricular chamber geometry and cardiac load which can modulate in vivo cardiac performance. At baseline, diabetic groups showed impaired contraction and relaxation function, as observed in our previous studies [26, 28]. As systolic blood pressure was slightly higher in diabetic groups, we could not rule out some influence from increased blood pressure on impaired ventricular function in diabetic rats. Therefore, myocardial function evaluation using papillary muscle preparations was important to reinforce the fact that systolic and diastolic function was depressed in diabetic rats.

Apocynin did not change cardiac structures or myocardial and ventricular function in the normoglycemic rats. Apocynin did increase E/A ratio in diabetic rats suggesting attenuation of diastolic dysfunction. In the papillary muscle preparations, developed tension was lower in DM than CTL after all positive inotropic stimulation. However, in DM + APO, values for developed tension were between those of CTL + APO and DM and did not significantly differ from either group. Therefore, the results from myocardial and ventricular functional evaluation suggest that apocynin slightly attenuated systolic and diastolic dysfunction in diabetic rats. Attenuation in myocardial interstitial fibrosis by apocynin in DM + APO may have contributed to these results. In fact, in other experimental model, such as the spontaneously hypertensive rat, development of myocardial fibrosis has been associated with impaired contractile function and heart failure development [57].

Unexpectedly, NADPH oxidase activity did not differ between groups. Increased NADPH oxidase activity in diabetic rat myocardium has previously been reported [13, 58–61]. These divergent results may have occurred due to different animal and experimental models, and NADPH oxidase activity analytical technique [59–61].

Reactive oxygen species can be neutralized by endogenous antioxidant systems that include GSH-Px, SOD, and catalase antioxidant enzymes. Despite no differences in NADPH oxidase activity, systemic antioxidant enzymes were modulated by apocynin and diabetes. Catalase, SOD, and GSH-Px activity was reduced in the DM group. The influence of apocynin differed between control and diabetic rats. Apocynin reduced SOD and GSH-Px activity in control rats, and restored catalase and SOD activity in diabetic rats. We have previously observed that apocynin reduces myocardial GSH-Px activity in hypertensive rats [20]. Apocynin-induced normalization of SOD activity was recently reported in diabetic rabbits [25]. Reactive oxygen species generation intensity modulates the response to oxidative stress. While low concentrations of reactive oxygen species stimulate antioxidant enzymes, high concentrations inhibit enzymatic activity leading to cellular damage [62]. Our results suggest that apocynin had a beneficial effect on diabetic rats by restoring SOD and catalase activity.

Apocynin (4-hydroxy-3-methoxyacetophenone) is a methoxy-substituted catechol derived from the medicinal herb *P. kurroa* [16]. Its antioxidant effects have been evaluated in several experimental studies, mostly as a NADPH oxidase inhibitor [17, 23]. However, this property has been put in check since apocynin failed to inhibit NADPH oxidase in cell culture; apocynin actually acted as a reactive oxygen species scavenger or a pro-oxidant agent [19, 63, 64]. Apocynin can also prevent NF-κB activation [24] and modulate nitric oxide-dependent pathways [65]. In several heart failure models, apocynin attenuated myocardial oxidative stress, apoptosis, hypertrophy, fibrosis, and diastolic dysfunction [11, 24, 66, 67].

However, only a few authors have examined the effects of apocynin on diabetic cardiomyopathy [13, 15, 25]. In accordance with our results, apocynin attenuated ventricular and myocardial dysfunction in diabetic mice [15, 49] and decreased reactive oxygen species production and apoptosis in the heart [13]. In diabetic mice [13, 15] and rabbits [25], apocynin also inhibited NADPH oxidase activity, which was not observed in our study. The conflicting result concerning these previous studies is probably related to the different animal species being evaluated. To the best of our knowledge, this is the first study to evaluate the cardiac effects of apocynin in diabetic rats. In summary, we showed that apocynin provided a mild improvement in ventricular diastolic function and myocardial contractile function and restored systemic SOD and catalase antioxidant enzyme activity. Further studies are needed to improve understanding of the influence apocynin has on the pathophysiology of diabetic cardiomyopathy.

## Conclusion

Apocynin restores serum antioxidant enzyme activity despite unchanged myocardial NADPH oxidase activity in diabetic rats. This study reinforces the potential beneficial influence of antioxidants in diabetic cardiomyopathy.

## Abbreviations

APO: apocynin
BW: body weights
[Ca^2+^]^0^: extracellular Ca^2+^ concentration
DHE: dihydroethidium
DM: diabetes mellitus
DT: peak of developed tension
+ dT/dt: maximum rate of tension development
− dT/dt: maximum rate of tension decline
EOH: 2-hydroxyethidium
E wave: early diastolic mitral inflow velocity
E/A: ratio between early (E)-to-late (A) diastolic mitral inflow
EF: ejection fraction
EFS: endocardial fractional shortening
GSH-Px: glutathione peroxidase
SOD: superoxide dismutase
IVRT: isovolumetric relaxation time
LA: left atrium
L_max_: peak of the length-tension curve
LV: left ventricle
LVDD: left ventricle diastolic diameter
LVSD: left ventricle systolic diameter
NADPH: nicotinamide adenine dinucleotide phosphate
PWSV: posterior wall shortening velocity
PWT: left ventricle diastolic posterior wall thickness
ROS: reactive oxygen species
RT: resting tension
S′: mitral annulus systolic velocity
SAP: systolic arterial pressure
TDI: tissue Doppler imaging
TPT: time to peak tension
